# Sulfosuccinimidyl oleate sodium is neuroprotective and alleviates stroke-induced neuroinflammation

**DOI:** 10.1186/s12974-017-1010-7

**Published:** 2017-12-04

**Authors:** Hiramani Dhungana, Mikko T. Huuskonen, Merja Jaronen, Sighild Lemarchant, Humair Ali, Velta Keksa-Goldsteine, Gundars Goldsteins, Katja M. Kanninen, Jari Koistinaho, Tarja Malm

**Affiliations:** 0000 0001 0726 2490 grid.9668.1 A.I. Virtanen Institute for Molecular Sciences, University of Eastern Finland, P.O. box 1727, FI-70211 Kuopio, Finland

**Keywords:** Stroke, CD36, SSO, Inflammation, Microglia, Neuroprotection

## Abstract

**Background:**

Ischemic stroke is one of the main causes of death and disability worldwide. It is caused by the cessation of cerebral blood flow resulting in the insufficient delivery of glucose and oxygen to the neural tissue. The inflammatory response initiated by ischemic stroke in order to restore tissue homeostasis in the acute phase of stroke contributes to delayed brain damage.

**Methods:**

By using in vitro models of neuroinflammation and in vivo model of permanent middle cerebral artery occlusion, we demonstrate the neuroprotective and anti-inflammatory effects of sulfosuccinimidyl oleate sodium (SSO).

**Results:**

SSO significantly reduced the lipopolysaccharide/interferon-γ-induced production of nitric oxide, interleukin-6 and tumor necrosis factor-α, and the protein levels of inflammatory enzymes including nitric oxide synthase 2, cyclooxygenase-2 (COX-2), and p38 mitogen-activated protein kinase (MAPK) in microglia, without causing cell toxicity. Although SSO failed to directly alleviate glutamate-induced excitotoxicity in murine cortical neurons, it prevented inflammation-induced neuronal death in microglia-neuron co-cultures. Importantly, oral administration of SSO in Balb/c mice subjected to permanent occlusion of the middle cerebral artery reduced microglial activation in the peri-ischemic area and attenuated brain damage. This in vivo neuroprotective effect of SSO was associated with a reduction in the COX-2 and heme oxygenase-1 immunoreactivities.

**Conclusions:**

Our results suggest that SSO is an anti-inflammatory and a possible therapeutic candidate in diseases such as stroke where inflammation is a central hallmark.

## Background

Ischemic damage following cerebral infarction is accompanied by a series of complex pathophysiological events including excitotoxicity, oxidative stress, inflammation, and apoptosis [1]. The post-ischemic inflammatory response in stroke is largely detrimental and contributes to the development of delayed brain damage [2]. Brain inflammation is mediated by microglia, astrocytes, and infiltrating peripheral leukocytes, primarily neutrophils and monocytes [3]. These inflammatory cells produce a variety of cytotoxic molecules, including pro-inflammatory cytokines, and reactive oxygen, and nitrogen species [4]. Microglia, in particular, are rapidly activated upon ischemia within hours of damage. Endogenous ligands including purines, peroxiredoxins, heat shock proteins, and high mobility group box-1 released from dying cells during ischemia bind to microglial surface receptors like cluster of differentiation 36 (CD36) and Toll-like receptors (TLRs) [5]. Intracellular signaling that follows the ligand-receptor interaction releases pro-inflammatory mediators like interleukin (IL)-1, IL-6, tumor necrosis factor-α (TNFα), cyclooxygenase-2 (COX-2), and nitric oxide synthase 1 (NOS1) that can damage the brain by further promoting inflammation and apoptosis. In contrast, anti-inflammatory cytokines like IL-10 and transforming growth factor-β (TGF-β) are involved in the resolution of inflammation [2]. Drugs, such as curcumin [6] and minocycline [7, 8], that alleviate inflammatory reactions have been shown to reduce ischemic damage and aid in functional recovery in preclinical models of stroke. Despite the lack of translational success and efficacy of anti-inflammatory agents thus far in human stroke [9, 10], modulation of microglial activation still holds promise in the treatment of stroke.

Sulfosuccinimidyl oleate sodium (SSO) is a long chain fatty acid that inhibits fatty acid transport into cells. SSO is known to bind the CD36 receptor on the surface of microglia. CD36 activation is involved in many inflammatory pathways and contributes to cell or tissue damage through production of free radicals, cytokines, and chemokines [11–14]. However, the role of SSO in inhibiting inflammation either directly or independently of CD36 in ischemic stroke has not been reported. We thus aimed to investigate whether SSO is able to attenuate stroke-related neuroinflammation in vitro and in vivo and provide benefit in a mouse model of ischemic stroke. We demonstrate for the first time that SSO inhibits key inflammatory mediators both in vitro and in vivo and that this anti-inflammatory effect is mediated by inhibition of phosphorylated p38 mitogen-associated protein kinase. Our data show that SSO provides neuroprotection against ischemic insult and can be a candidate molecule for further drug development.

## Methods

### BV2 cell culture

The mouse BV2 microglial cell line was kindly provided by professor Rosario Donato (University of Perugia, Italy). Cells were maintained in a humidified chamber with 5% CO2 and 37 °C in RPMI-1620 medium (Invitrogen, CA, USA) supplemented with 10% heat-inactivated fetal bovine serum (FBS, Invitrogen) and 0.6 μg/ml gentamicin (Sigma-Aldrich, MO, USA). Cells were seeded at a density of 1.5 × 10^5^ cells/ml for both protein and RNA extraction. Culture media was replaced by serum-free RPMI media 24 h after seeding. On the next day, the cells were stimulated with 100 ng/ml lipopolysaccharide (LPS, Sigma-Aldrich) and 5 ng/ml interferon-γ (IFN-γ, Peprotech, NJ, USA) and co-treated with SSO (TRC, Toronto, Canada) for a further 24 h.

### Primary cortical neuron cultures

Primary neuronal cultures were prepared as described previously [15]. Briefly, cortical tissue from E15 C57BL/6J embryos was dissected out after carefully removing the meninges. The dissected cortices were transferred to Krebs buffer (0.126 M NaCl, 2.5 mM KCl, 25 mM NaHCO_3_, 1.2 mM NaH_2_PO_4_, 1.2 mM MgCl_2_, 2.5 mM CaCl_2_, pH 7.4) containing 0.025% trypsin and incubated at 37 °C for 15 min. Then, the reaction was stopped by using trypsin inhibitor containing DNase (Sigma-Aldrich). Neurons were dissociated, counted, and plated in poly-l-lysine (Sigma-Aldrich)-coated 48-well plates at a density of 1.25 × 10^5^ cells/well in neurobasal media (Gibco, NY, USA) supplemented with 2% B27 (Thermo Fisher Scientific, MA, USA), 500 μM L-glutamine (Thermo Fisher Scientific), and 1% penicillin-streptomycin (P/S, Thermo Fisher Scientific). Fifty percent of the media was changed 5 days after seeding. At day 6, the neurons were exposed to 50 μM of SSO for 2 h and then 400 μM of glutamate in the presence of 50 μM SSO for 24 h.

### Primary microglia cultures

Primary microglia cultures were prepared as described previously [15]. Briefly, P0-P2 C57BL/6J mouse brains were dissected and rinsed in phosphate buffer solution (PBS) containing 1 g/l glucose. Brains were mechanically dissociated and digested using 0.5% trypsin-EDTA for 20 min at 37 °C. The tissue homogenate was suspended in DMEM/F12 (Gibco) containing 10% heat-inactivated FBS and 1% P/S (Thermo Fisher Scientific) and plated on 150-mm cell culture dishes for 3 weeks. After reaching confluency, astrocytes were removed using 0.25% trypsin in Hank’s Balanced Salt Solution (HBSS, Gibco) in 1:4 dilution in serum-free DMEM/F12. After careful washes with PBS, the microglia attached on the bottom of the plates were isolated by using 0.25% trypsin in PBS. Fresh culture medium containing FBS was added to neutralize the effect of trypsin. Cells were then centrifuged and plated for co-culture studies.

### Neuron-BV2 co-culture and neuron-primary microglia co-cultures

For primary neuron-BV2 co-culture, cortical neurons were plated onto a 48-well plate at a density of 1.25 × 10^5^ cells/well in neurobasal media as described previously. At day 5, BV2 cells were plated on top of neurons at a density of 1:5 (25,000 BV2 cells for 125,000 neurons) for 2 h. Cells were pre-treated with SSO for 2 h and then exposed to 100 ng/ml LPS and 5 ng/ml IFNγ for 48 h. The co-cultures were rinsed with PBS, fixed with 4% PFA for 20 min, and washed again with PBS. Neuronal viability in the co-cultures was evaluated as described previously [16]. The co-cultures were stained with microtubule-associated protein 2 (MAP-2) peroxidase labeling, and the ABTS peroxidase substrate kit (Vector) was used to develop the color for the analysis according to the manufacturer’s instructions. The green-colored product was formed after incubation for 30 min with ABTS kit in the dark. One hundred fifty microliter of the substrate solution was transferred to a 96-well plate, and the absorbance was measured using a microtiter plate reader Victor 2.0 (Perkin Elmer, MA, USA) at 405 nm. For primary neuron-microglia co-culture, cortical neurons were similarly plated onto 48-well plates at a density of 1.25 × 10^5^ cells/well. At day 5, primary microglia were plated on top of neurons in neurobasal media at a density of 1:2 (62,500 microglia for 125,000 neurons). On the next day, the cells were pre-treated with SSO for 2 h and then exposed to 100 ng/ml LPS and 30 ng/ml IFN-γ for 48 h. The cells were then rinsed with PBS, fixed with 4% PFA, and the neuronal viability assessed by ABTS peroxidase substrate kit as described above.

### Measurement of nitric oxide production and cell viability assay

Nitric oxide (NO) production was assessed in the culture media by using Griess assay 24 h after LPS/IFNγ stimulation and SSO treatment. Fifty microliter of culture supernatant was incubated with an equal volume of Griess reagent for 10 min at room temperature (RT), and the optical density was measured at 544 nm using Victor 2.0 plate reader.

Cell viability was measured using resazurin assay 24 h after exposure. Briefly, cells were incubated with 10 μM resazurin (Sigma-Aldrich) diluted in culture media for 2 h at 37 °C. The absorbance was then quantified at 485 nm using Victor 2.0 plate reader.

### CBA assay

Culture supernatants obtained 24 h after SSO treatment were used to determine the levels of interleukin-6 (IL-6), IL-10, monocyte chemoattractant protein 1 (MCP-1), TNF-α, IFN-γ, and IL-12p70 using a mouse anti-inflammatory cytometric bead array (CBA) kit (BD Biosciences, CA, USA). After staining, the samples were run on a FACS Calibur flow cytometer (BD Biosciences). The results were analyzed using FCAP array software (Soft Flow Hungary Ltd., Pecs, Hungary).

### Western blot

Twenty-four hours after SSO treatment, BV2 cells were washed with PBS and collected in SDS sample buffer (0.0625 M TRIS-HCl, 2.3% SDS, 5% β-mercaptoethanol, 10% glycerol, bromophenol blue). For analyzing the nuclear localization of NFκB, BV2 cells were treated as described above and subcellular fractions were generated with Cell Fractionation kit (Cell Signaling) according to the manufacturer’s protocol. Ten micrograms of protein were loaded and run on 10% SDS-PAGE gels. The proteins were transferred onto PVDF-hybond membranes for 1 h at 4 °C. The membranes were then blocked in 5% milk in 0.2% PBS-tween (PBST) for 30 min. Primary antibodies for total cell lysates (nitric oxide synthase 2 (NOS2), 1:2000 dilution, EMD Millipore, MA, USA; COX-2, 1:1000 dilution, BD Biosciences; heme oxygenase-1 (HO-1), 1:1000 dilution, ENZO Lifesciences, NY, USA; P-p38, 1:1000 dilution, Cell Signaling Technology, MA, USA) and for nuclear fractions (NFκB p65, 1:1000 dilution, Santa Cruz, TX, USA) in 5% BSA in PBS and 0.02% NaN3 were incubated with the membranes overnight at 4 °C. After washing with PBST, appropriate secondary antibodies were incubated for 2 h at RT diluted at 1:2000 in a blocking solution (anti-mouse ECL-HRP; GE healthcare life science, Uppsala, Sweden, or anti-rabbit IgG HRP Conjugate, Bio-Rad, CA, USA). The membranes were washed again with PBST, and the protein bands were visualized using an ECL-plus kit (Thermo Fisher Scientific) under Storm 860 scanner (Molecular dynamics, Caesarea, Israel). β-actin (1:2000 dilution, Sigma-Aldrich) and histone-3 (H3) (1:1000 dilution, Abcam, Cambridge, UK) antibodies were used as loading controls for total cell lysates and nuclear fractions, respectively. Their immunoreactivities were detected with Cy3-conjugated anti-rabbit secondary antibody (Jackson ImmunoResearch Laboratories, PA, USA) and scanned with G:BOX imaging system (Syngene, Cambridge, UK). Densitometric analysis was performed using ImageQuant TL software (GE healthcare life science).

### Quantitative real-time PCR

The relative expression levels of IL-6, NOS2, nuclear factor erythroid 2-related factor-2 (Nrf2), and HO-1were measured in BV2 cells exposed to LPS/IFNγ and SSO using quantitative PCR (qPCR). Total RNAs were isolated using the RNeasy mini kit (Qiagen, CA, USA) according to the manufacturer’s instructions. The concentration and purity of RNA samples were determined using a NanoDrop 2000 (Thermo Fisher Scientific). Complementary DNA (cDNA) was synthesized by using 500 ng of total RNA, Maxima Reverse Transcriptase, dNTP, and random hexamer primers (Life Technologies, CA, USA). The final concentration of cDNA was 2.5 ng/μl. qPCR was run using the StepOne Plus Real-Time PCR system (Applied Bioscience, CA, USA). Gene expression analysis was carried out using comparative C_t_ method (△△C_T_) where the threshold cycle of the target genes was normalized to glyceraldehyde 3-phosphate dehydrogenase (GAPDH) and ribosomal RNA internal housekeeping gene controls (△C_T_). The mRNA expression is presented as a fold change.

### Animals

A total of 21 4-month-old male BALB/cABom mice (Taconic, Köln, Germany) obtained by breeding in-house were used in this study. The mice were housed under a 12-h light/dark cycle and allowed for food and water ad libitum. All the animal experiments were approved by the National Animal Experiment Board of Finland and followed the Council of Europe Legislation and Regulation for Animal Protection. The animals were randomized and divided into two groups, SSO treated (*n* = 10) and control (*n* = 11) using GraphPad QuickCalcs software (GraphPad Software, Inc. La Jolla, CA, USA).

### Ischemia surgery and treatment with SSO

All animals underwent distal permanent occlusion of the middle cerebral artery (MCA) (pMCAo) as described previously [17]. Briefly, mice were anesthetized with 5% isoflurane for induction and 2% isoflurane for maintenance (70% N_2_O/30% O_2_). The temperature of the mice was maintained at 36-5 ± 0.5 °C using a thermostatically controlled heating blanket connected to a rectal probe (PanLab, Harvard Apparatus, Barcelona, Spain). The temporalis muscle was retracted to expose the skull in between the ear and the eye, and a small hole of approximately 1 mm was drilled at the site of the MCA. The dura was carefully removed to expose the MCA. The artery was then gently lifted up and cauterized using a thermocoagulator (Bovie Medical Corporation, Clearwater, FL, USA). After the procedure, the muscle was lifted back and the skin wound was sutured. The animals were then placed back to their home cages. SSO was emulsified in 0.5% methyl cellulose and administered once by single oral gavage at the dose of 50 mg/kg immediately after the surgery, when the mice had retained their consciousness. The administration routes for SSO in vivo have been described previously [18, 19]. In addition, we performed a dose-response study and found that SSO at 50 mg/kg was suitable to see a beneficial effect after stroke. There was no mortality in this study.

### Magnetic resonance imaging

Infarct size was imaged by magnetic resonance imaging (MRI) at 3 days post-injury using a vertical 9.4T Oxford NMR 400 magnet (Oxford Instrument PLC, Abingdon, UK). Mice were anesthetized and maintained at 1.5% isoflurane during the imaging. A quadrature volume radiofrequency coil was used for transmission and reception. Multislice T2-weighted images (repetition time 3000 ms, echo time 40 ms, matrix size 128 × 256, field of view 19.2 × 19.2 mm^2^, slice thickness 0.8 mm, and number of slices 12) were taken with double spin-echo sequences with adiabatic refocusing pulses. Lesion volume was outlined manually on T2-weighted images using an in-house software (Aedes) under MATLAB (Math-works, Natick, MA, USA) environment. The total lesion volume and the volume of the left and right hemispheres were calculated by multiplying the number of pixels with the pixel size and slice thickness. The relative percentage of the infarction volume was calculated as described previously: infarction volume = (volume of left hemisphere − (volume of right hemisphere − measured infarct volume))/volume of left hemisphere [20]. The analysis was performed blinded to the study groups.

### Perfusion, blood sampling, and immunohistochemistry

All mice were sacrificed immediately after MRI imaging at 3 days post-injury. The mice were anesthetized using 250 mg/kg of avertin and transcardially perfused with heparinized saline (2500 IU/l). The brains were removed and post-fixed in 4% paraformaldehyde (PFA) for 24 h followed by cryoprotection in 30% sucrose for 48 h. The brains were snap frozen in liquid nitrogen and cut into 20-μm-thick serial coronal sections at the start of the lesion using a cryostat (Leica Microsystems GmbH, Wetzlar, Germany).

For the detection of microglia, astrocytes, COX-2, and HO-1 immunoreactivity, a set of six consecutive sections at 400 μM intervals starting from the beginning of the lesion were used. The sections were incubated against primary antibody ionized calcium binding adapter molecule 1(Iba-1, 1:250 dilution, Wako Chemical GmbH, Neuss, Germany), CD45 (1:100 dilution, BioRad, Hercules, CA, USA), NeuN (1:200 dilution, EMD Millipore, Temecula, CA, USA), glial fibrillary acidic protein (GFAP, 1:500 dilution; DAKO; Glostrup, Denmark or 1:200 dilution; EMD Millipore), COX-2 (1:500 dilution; Cayman Chemicals, Ann Arbor, MI, USA), and HO-1 (1:1000, ENZO) overnight at RT. The sections were then incubated with biotinylated secondary antibody (1:200 dilution, Vector, Burlingame, CA, USA) for 2 h and then avidin-biotin complex reagent (Vector) according to the manufacturer’s instructions. Alternatively, Alexa 568-conjugated secondary antibody (1:200 dilution; Invitrogen, Eugene, OR, USA) was used. Immunoreactivities were visualized by the development with nickel-enhanced 3, 3′-diaminodenzidine.

Immunoreactivities in the peri-ischemic area adjacent to the infarct border were imaged at ×10 magnification using AX70 microscope (Olympus Corporation, Tokyo, Japan) with an attached digital camera (Color View 12 of F-view, Soft Imaging System, Munster, Germany) and a computer running analysis software (Soft imaging system). The intensity of the immunoreactivities was quantified from an area of 718 × 532 μm adjacent to the infarct border using ImagePro Software (Media Cybernetics, Silver Spring, MD, USA) blinded to the study groups. A total of seven to ten mice per group were analyzed.

### Statistical analysis and exclusion criteria

Statistical analysis was performed using GraphPad Prism (GraphPad Software Inc., CA, USA) by running one-way ANOVA with Tukey’s post hoc test or student *t* test where appropriate. Statistical outliers were excluded from the data set after performing Grubb’s test using GraphPad Prism QuickCalcs. All the data are expressed as mean ± SEM and *P* < 0.05 was used for the determination of statistical significance.

## Results

### SSO maintains the viability of BV2 microglia upon inflammatory stimuli

**Fig. 1:**
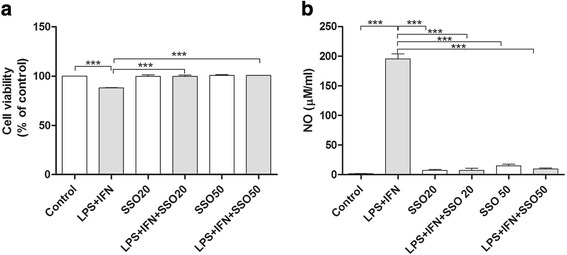
SSO prevented inflammation-induced cell viability alteration and NO release. SSO alone did not alter the viability of BV2 cells. Exposure of BV2 cells to 100 ng/ml LPS and 5 ng/ml IFNγ significantly reduced the viability of BV2 cells while simultaneous treatment with SSO prevented it as revealed by resazurin assay (**a**). The values are normalized to control. In addition, LPS/IFNγ treatment induced a massive NO production in BV2 cells which was blocked by co-treatment with SSO at both concentrations as measured by Griess reaction (**b**). The results were analyzed with one-way ANOVA followed by Tukey’s post hoc test. Data are expressed as mean ± SEM. *n* = 3 per group from three independent experiments. *p < 0.01, **p < 0.01 and ***p < 0.001

The ability of SSO to alter cell viability was first assessed with naïve BV2 cells or BV2 cells stimulated with 100 ng/ml LPS and 5 ng/ml IFNγ. Two concentrations of SSO (20 μM and 50 μM) were used. SSO alone did not alter the cellular viability as measured by the resazurin assay (Fig. 1a). Exposure to 100 ng/ml LPS + 5 ng/ml IFNγ modestly, yet significantly reduced the viability of the BV2 cells. Co-treatment with SSO prevented the LPS + IFNγ-induced reduction in the cell viability (Fig. 1a). In addition, LPS + IFNγ exposure induced a massive NO production in BV2 cells which was blocked by co-treatment with SSO at both concentrations (Fig. 1b).

#### SSO downregulates LPS/IFNγ-induced inflammatory mediators in BV2 cells

**Fig. 2:**
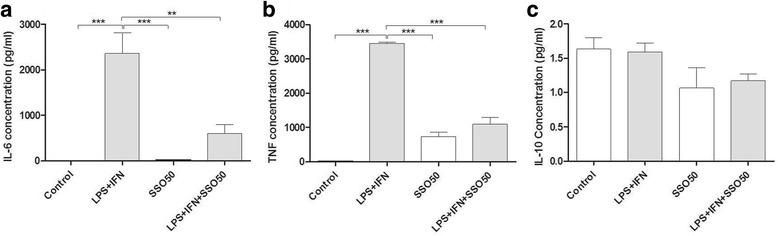
SSO reduced the concentration of IL-6 and TNF-α secreted into the culture media in LPS + IFNγ-stimulated BV2 cells. Culture media were collected from BV2 cells stimulated with LPS + IFNγ for 24 h in the presence or absence of SSO. The collected media were analyzed for secreted levels of IL-6 (**a**), TNF-α (**b**), and IL-10 (**c**) using the CBA. The results were analyzed with one-way ANOVA followed by Tukey’s post hoc test. Data are expressed as mean ± SEM. *n* = 3–4 per group derived from independent experiments. *p < 0.01, **p < 0.01, and ***p < 0.001

Since 50 μM dose of SSO did not exert any toxicity, we chose to use SSO at the 50 μM concentration for further experiments. Next analyses focused on analyzing whether SSO exhibits anti-inflammatory properties in vitro in LPS + IFNγ-stimulated BV2 cells. Of the various cytokines analyzed, LPS + IFNγ promoted a massive increase in the secretion of IL-6 and TNF-α, which were significantly reduced by co-treatment with SSO (Fig. 2a, b). The levels of IL-10 were unaltered in all treatment groups (Fig. 2c).

**Fig. 3:**
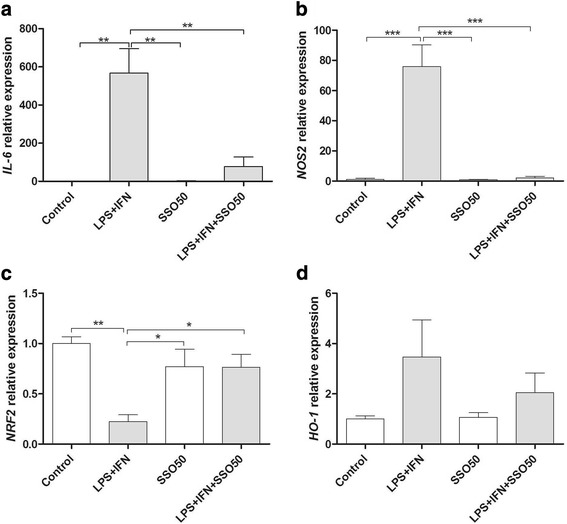
SSO reduced the LPS + IFN-induced pro-inflammatory mediators *IL-6* and *NOS2* while increasing *Nrf2*, the main regulator of the antioxidant response. BV2 cells were exposed to LPS + IFNγ in the presence or absence of SSO for 24 h. The mRNA expression levels of *IL-6* (**a**), *NOS2* (**b**), *Nrf2* (**c**), and *HO-1* (**d**) were analyzed by qRT-PCR. Results are expressed as mean ± SEM. The results were analyzed with one-way ANOVA followed by Tukey’s post hoc test. Data are expressed as mean ± SEM. *n* = 3 per group from three independent experiments, and the values were normalized to controls. *p < 0.01, **p < 0.01, and ***p < 0.001

We next analyzed whether SSO influences the expression of genes involved in the inflammation and redox signaling. As expected, LPS + IFNγ significantly increased the mRNA expression of *IL-6* (Fig. 3a) and *NOS2* (Fig. 3b). Co-treatment with SSO blocked the increase in the expression levels of both *IL-6* and *NOS2*. Interestingly, LPS + IFNγ reduced the expression levels of *Nrf2* which was returned to basal levels by co-treatment with SSO (Fig. 3c). In contrast, SSO treatment alone or in combination with LPS + IFNγ did not affect the levels of heme oxygenase 1 (*HO-1*), one of the well-known targets genes of *Nrf2*(Fig. 3d).

**Fig. 4:**
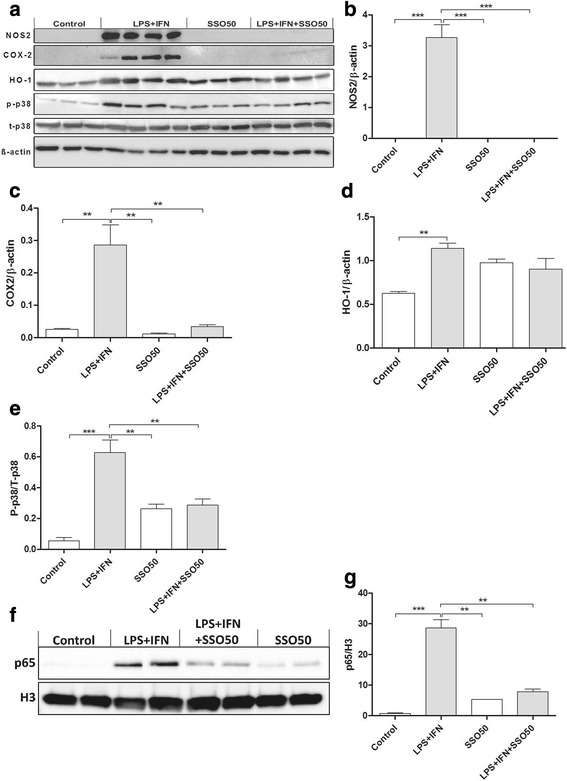
SSO reduces the protein levels of activated p38, iNOS, and COX-2 in vitro*.* BV2 cells were exposed to 100 ng/ml of LPS and 5 ng/ml of IFNγ in the presence or absence of 50 μM SSO. Figure (**a**) shows the representative images of Western blots probed for NOS2, COX-2, HO-1, p-p38, t-p38, and β-actin as control. Stimulation of BV2 cells with LPS/IFNγ for 24 h drastically increased the levels of NOS2 (**b**), COX-2 (**c**), HO-1 (**d**), and P-p38/T-p38 (**e**). Representative image of nuclear NFκB p65 translocation after nuclear fractionation. Nuclear-specific marker H3 was used to normalize p65 translocation (**f**). LPS/IFNγ stimulation resulted in robust nuclear translocation of NFκB p65, which was significantly prevented by SSO co-treatment (**g**). The results were analyzed with one-way ANOVA followed by Tukey’s post hoc test. Data are expressed as mean ± SEM. *n* = 3-4 per group from independent experiments. *p < 0.01, **p < 0.01, and ***p < 0.001

To further establish the anti-inflammatory properties of SSO, we measured the protein levels of COX-2 and NOS2, which are responsible for the production of NO and pro-inflammatory mediators. Figure 4a shows a representative Western blot analysis which revealed that SSO co-treatment significantly reduced the LPS + IFNγ-induced expression of NOS2 and COX-2 in BV2 cells (Fig. 4b, c). Furthermore, SSO alone increased the basal protein levels of HO-1 but did not alter the LPS + IFNγ-induced levels of HO-1 (Fig 4d). Since both LPS and IFNγ are known to increase the production of NOS2 through activation of MAPK pathway, we analyzed the protein expression levels of the activated p38. Western blot analysis revealed a significant LPS/IFNγ-induced upregulation in the phosphorylated form of the p38, which was prevented by co-treatment with SSO (Fig. 4e). We observed no changes in the levels of phosphorylated JNK and ERK between treated and untreated groups (data not shown).

Furthermore, p38 MAPK signaling pathway is known to increase nuclear translocation of NFκB p65, whereas inhibition of CD36, especially by SSO, is known to block this translocation especially after ceramide stimulation in pancreatic beta cells [21]. This was confirmed in our in vitro model, where we were able to demonstrate a robust nuclear translocation of NFκB p65 upon LPS/IFNγ exposure, which was significantly alleviated by SSO treatment (Fig. 4f, g).

### SSO rescues neurons from inflammation-induced death

**Fig. 5:**
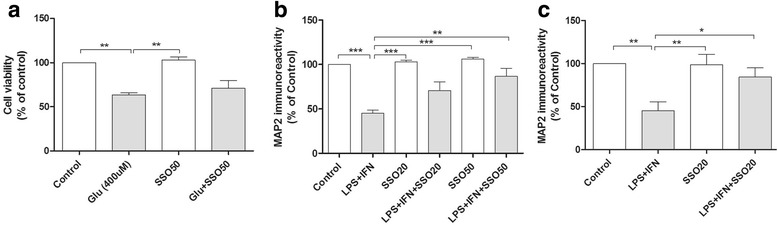
SSO prevented inflammation-induced neuronal death in microglia/neuron co-culture. SSO was not directly protective against glutamate-mediated neurotoxicity. Primary neurons were pre-exposed to 50 μM SSO for 2 h followed by subsequent exposure to 400 μM glutamate and 50 μM SSO for 24 h. Cell viability was assayed using the MTT assay (**a**). SSO prevented inflammation-induced neuronal death in neuron-glial co-cultures. The co-cultures were pre-treated with two concentrations of SSO (20 μM and 50 μM) for 2 h followed by the exposure to LPS + IFNγ in the presence or absence of SSO. MAP-2 immunoreactivity was measured to determine neuronal viability using peroxidase-ABTS kit. SSO prevented the LPS + IFNγ-induced neuronal death in neuron/BV2 co-cultures (**b**) and neuron/primary microglia co-cultures (**c**). Data are expressed in mean ± SEM and statistical analysis carried out using one-way ANOVA followed by Tukey’s post hoc test. *n* = 3 per group from three independent experiments, and the values are normalized to controls. *p < 0.01, **p < 0.01, and ***p < 0.001

We next assessed whether SSO is directly neuroprotective in cultured primary neurons exposed to glutamate-mediated excitotoxicity. Primary neurons were pre-treated with 50 μM SSO for 2 h followed by the exposure to 400 μM glutamate and 50 μM SSO for 24 h. SSO alone was not toxic to the neurons, yet it was unable to prevent glutamate-induced neuronal death (Fig. 5a). Since SSO was not able to rescue neuronal viability in pure neuronal cultures but has anti-inflammatory properties, we assessed whether SSO can prevent inflammation-induced neuronal death using primary neuron-BV2 co-cultures. The co-cultures were pre-treated with two concentrations of SSO (20 μM and 50 μM) followed by the exposure to 100 ng/ml LPS and 5 ng/ml IFNγ. Measurement of neuronal viability using peroxidase-ABTS kit showed that SSO alone was not causing any loss of MAP-2 immunoreactivity. Moreover, SSO dose-dependently prevented the LPS/IFNγ-induced neuronal death (Fig. 5b). To confirm these findings on primary microglia, we exposed a primary neuron-primary microglia co-culture model to 20 μM SSO, and similarly, 2-h SSO pre-treatment significantly prevented LPS + IFNγ-induced neuron death (Fig. 5c).

### SSO treatment attenuates brain damage following ischemia

**Fig. 6:**
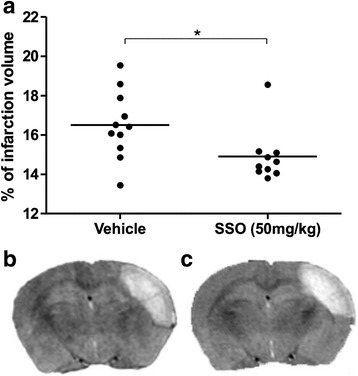
SSO-treated mice show attenuated infarct size. Quantification of MRI images taken at 3 days post-injury revealed that oral administration of SSO reduced brain damage following ischemia (**a**). Data are expressed as mean ± SEM. Vehicle-treated mice (*n* = 11) and SSO-treated mice (*n* = 10). *p < 0.05. Figure (**b, c**) represents the typical T2 weighted image of vehicle- (**b**) and SSO-treated mice (**c**)

Based on our in vitro data, we then tested the therapeutic effect of SSO in a mouse model of pMCAo. We chose the dose of 50 mg/kg of SSO, which caused no adverse effect to the mice. The mice underwent MRI imaging at 3 days post-injury. Quantification of the lesion volumes revealed that orally administered SSO significantly reduced the cortical ischemic infarct size compared to vehicle-treated controls (Fig. 6).

### Peri-ischemic microgliosis but not astrogliosis was significantly reduced in SSO-treated mice

**Fig. 7:**
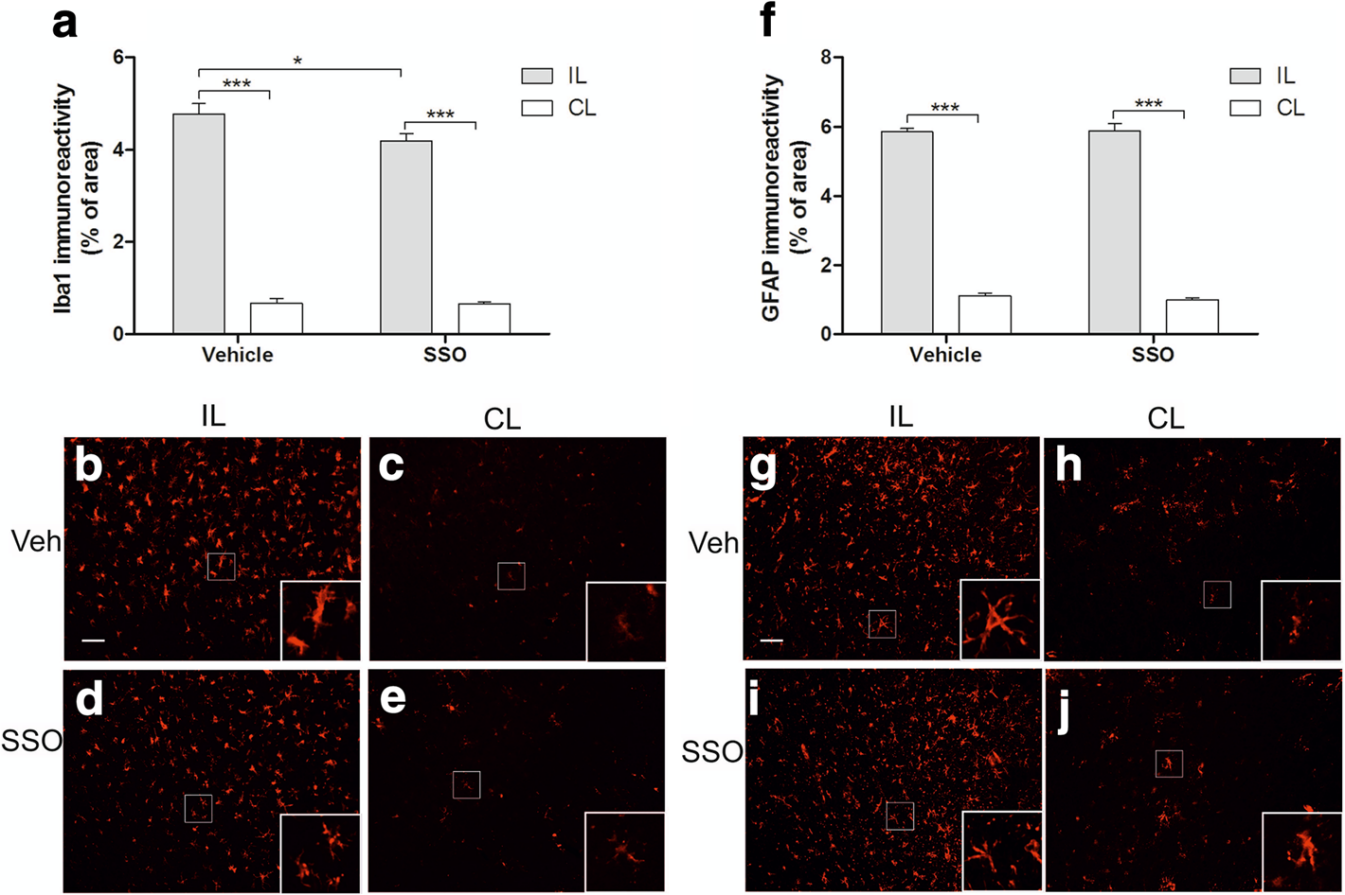
Brain microgliosis is reduced upon treatment with SSO. GFAP and Iba-1 immunoreactivities were quantified at 3 days post-injury. Quantification of Iba-1 immunoreactivity (**a**) and representative images of Iba-1 staining in the peri-ischemic area of the ipsilateral (**b**) and contralateral hemisphere (**c**) of vehicle-treated mouse. The lower panels (**d**, **e**) represent the corresponding areas in SSO-treated mouse. Quantification of GFAP immunoreactivity (**f**) and the representative images from the peri-ischemic area of the ipsilateral (**g**) and contralateral (**h**) hemisphere in vehicle-treated mouse. Panels **i** and **j** represent corresponding areas in the SSO-treated mouse. High magnification insets show GFAP and Iba-1 positive cell morphologies. Data are expressed as mean ± SEM. GFAP immunoreactivity was quantified from vehicle-treated (*n* = 10) and SSO-treated (*n* = 9) mice. Iba- 1 immunoreactivity was quantified from vehicle-treated (*n* = 10) and SSO-treated (*n* = 10) mice. *p < 0.05 and ***p < 0.001. *IL* ipsilateral, *CL* contralateral

Ischemia-induced brain microgliosis was analyzed by immunohistochemical staining against Iba-1. As expected, we detected a significant upregulation of microgliosis in the peri-ischemic area of both the SSO-treated and control mice when compared to the corresponding area in the contralateral side at 3 days post-stroke (Fig. 7a). However, the extent of Iba-1 immunoreactivity in the peri-ischemic area of SSO-treated mice was significantly reduced compared to their vehicle-treated counterparts (Fig. 7a). Ischemia induced significant upregulation of GFAP immunoreactivity in the peri-ischemic area compared to the contralateral side, yet SSO failed to reduce stroke-induced increased GFAP immunoreactivity (Fig. 7b).

### SSO-treated mice showed reduced expression of COX-2 and increased expression of HO-1 in the peri-ischemic area

**Fig. 8:**
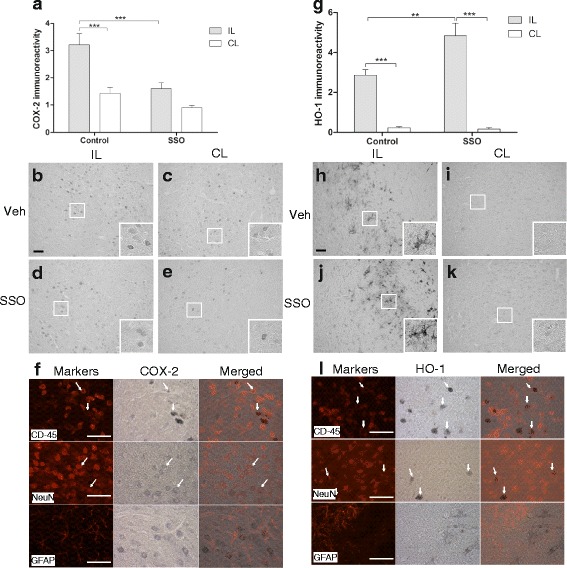
COX-2 immunoreactivity was decreased while HO-1 immunoreactivity was increased in SSO-treated mice at 3 days post-stroke. pMCAo induced a significant increase in the levels of COX-2 immunoreactivity. COX-2 immunoreactivity was significantly reduced in SSO-treated mice compared to vehicle-treated mice (**a**). Representative images of COX-2 immunoreactivity in the peri-ischemic area of the ipsilateral (**b**) and contralateral side (**c**) of vehicle-treated mouse. Panels (**d** and **e**) represent the corresponding areas for the SSO treated mouse. HO-1 immunoreactivity was increased in SSO-treated mice when compared to vehicle-treated mice (**g**). Panels **h** and **i** show representative images of HO-1 immunoreactivities in the peri-ischemic ipsilateral and contralateral side of the vehicle-treated mouse, respectively. Panels **j** and **k** represent the corresponding areas in the SSO-treated mice, respectively. High magnification insets show the cellular morphology of the staining. Panels **f** and **l** show double staining of COX-2 (**f**) and HO-1 (**l**) together with markers of microglia/macrophage (CD45), neurons (NeuN), and astrocytes (GFAP) to confirm the cell types expressing these inflammatory mediators. Scale bar = 50 μm. Data are expressed as mean ± SEM. Immunoreactivities were quantified from the total of seven to ten mice per group. **p < 0.01 and ***p < 0.001. *IL* ipsilateral, *CL* contralateral

Due to the ability of SSO to reduce the expression of COX-2 in vitro, we analyzed the extent of COX-2 immunoreactivity in the peri-ischemic area of the stroked animals at 3 days post-stroke. pMCAo induced a significant upregulation in COX-2 immunoreactivity in the peri-ischemic area (Fig. 8a). SSO-treated animals showed a reduced extent of COX-2 expression compared to vehicle-treated controls (Fig. 8a). To evaluate the cell types expressing COX-2, we carried out immunohistological double stainings with COX-2 and microglial/macrophage marker CD45, neuronal marker NeuN, and astrocytic marker GFAP. The double stainings revealed that COX-2 immunoreactivity was mainly localized in microglia/macrophages and neurons but not in astrocytes (Fig. 8f). To evaluate the impact of SSO to induce antioxidant response, we carried out a staining against HO-1 and detected a significant upregulation in HO-1 in the peri-ischemic area in the SSO-treated animals when compared to vehicle-treated controls (Fig. 8g). Double stainings of HO-1 with CD45, NeuN, and GFAP revealed colocalization similar to COX-2, HO-1 was mainly expressed in microglia/macrophages and neurons, but not in astrocytes (Fig. 8l).

## Discussion

Here, we show for the first time that oral administration of SSO is neuroprotective in a mouse model of ischemic stroke. SSO-induced neuroprotection was associated to decreased levels of pro-inflammatory mediators and increased levels of the antioxidant HO-1. The neuroprotective effect of SSO was related to its anti-inflammatory activity, since SSO failed to directly prevent glutamate-induced neuronal death, yet it preserved microglia viability; attenuated the production of inflammatory mediators NO, IL-6, and TNF-α upon the exposure to LPS + IFNγ; and prevented inflammation-induced neuronal death in vitro.

Inflammation plays a pivotal role in many cerebral injuries and diseases including stroke. Stroke induces rapid microglial activation and promotes the secretion of a variety of inflammatory mediators including IL-1β, TNF-α, and IL-6 [22]. In addition, inflammatory enzymes like NOS, COX-2, and MAP kinases are rapidly upregulated, further exaggerate the inflammatory response and induce neuronal death. The pro-inflammatory events occurring in the ischemic brain can be modeled in vitro by exposing microglia to LPS. LPS binds to Toll-like receptors (TLRs), such as TLR4, and initiates the activation of the p38 MAPK and NFκB pathway resulting in the production of NOS2, NO, and various pro-inflammatory cytokines, including IL-6 and TNF-α [23–25]. NO, IL-6, and TNF-α are classical TLR4-regulated pro-inflammatory mediators known to be induced in ischemic stroke and propagate apoptosis and the secondary ischemic damage mediated by [26, 27]. TLR4 deficiency reduces the levels of NO, IL-6, TNF-α and protects against ischemic-induced neuronal damage [9, 26, 28]. In accordance, inhibition of p38 MAPK pathway has been reported to be protective in both transient and permanent focal cerebral ischemia [29–31]. Similarly, preventing the activation of the NFκB pathway is protective in both permanent and transient cerebral ischemia [32, 33]. Neuroprotective agents modulating inflammation such as curcumin [6], hydroxysafflor yellow A [34], and minocycline [7, 8] inhibit microgliosis and the activation of NFκB and p38 pathways. Thus, it is not surprising that the neuroprotective and anti-inflammatory effects of SSO were associated with diminished activation of these pathways. Moreover, NO is the key mediator of neuronal death in neuron-microglia co-culture model used in this study [16], and SSO inhibited the secretion of NO, together with IL-6 and TNF-α upon the exposure to LPS + IFNγ and microgliosis in vivo, thus supporting the potent anti-inflammatory capacity of SSO.

SSO has been shown to act as an inhibitor of CD36 in vitro [35–37]. CD36 is a scavenger receptor involved in pathogen recognition and phagocytosis and is abundantly expressed in microglia/macrophages. CD36 is bound by a wide variety of ligands to promote downstream signaling resulting in pro-inflammatory activation [5, 38] including the activation of the NFκB pathway [39, 40]. Indeed, CD36 signaling is induced after cerebral stroke and plays a pivotal role in stroke-induced inflammation [41]. Thus, it is not surprising that CD36-deficient mice show reduced levels of NFκB activation and oxidative stress which alleviates brain damage [39]. Cao et al. [42] have shown that CD36 participates in LPS-induced inflammation in goat mammary epithelial cells by regulating NFκB and JNK activation but not p38-MAPK pathway. It is therefore plausible that SSO binds to the active site of CD36 to exert neuroprotection. However, reduced p38 activation by SSO in the current study suggests that also other mechanisms may underlie the neuroprotective effect of SSO. In fact, the impact of CD36 downstream signaling may be model- and context-dependent. This hypothesis is supported by a study demonstrating an increase in the extent of injury in mice lacking CD36 after acute neonatal stroke [43] and on the other hand by a study in which genetic deletion of CD36 provided benefit in a transient model of ischemic stroke [13]. Moreover, Kim et al. reported that a specific tetrapeptide inhibitor for CD36, SS-31, or genetic deletion of CD36 is protective only in transient but not permanent MCAo [44]. Noteworthy is that the dose of SS-31 used by Kim et al. and SSO used in the current study cannot be compared also due to different administration routes and time points. Likewise, the permanent stroke model used in the current study (distal pMCAo) is different from that used by Kim et al., possibly explaining the differences in the outcome.

Our in vitro data are well in line with our in vivo data showing the ability of SSO to reduce microglial activation and the expression of COX-2. COX-2, an enzyme upregulated following cerebral ischemia, contributes to the ischemic brain injury [45]. CD36 deficiency has been shown to be associated with downregulation of COX-2 in a rodent model of age-related macular degeneration [46], and CD36 activation has been proposed to increase the expression of COX-2 [47]. In line with these data, we found an upregulation of COX-2 immunoreactivity in the peri-ischemic area after stroke which was attenuated in SSO-treated mice. COX-2 immunoreactivity was mainly co-localized in neurons and microglia/macrophages. In addition, our in vitro data also show a reduction in the protein levels of COX-2 in SSO-treated microglia following the exposure to LPS + IFNγ. CD36 activation has been shown to contribute to the generation of pro-inflammatory eicosanoids in vitro [48]. Thus, the reduction in COX-2 activity by SSO might restrict the formation of pro-inflammatory eicosanoids by binding to CD36 and hence reduced brain damage which is likely to be reflected as decreased COX-2 expression also in affected neurons.

HO-1 induction has been reported to be beneficial in cerebral stroke in many models. HO-1 is an endogenous antioxidant enzyme upregulated in cerebral ischemia and catalyzes the conversion of heme to carbon monoxide and biliverdin. HO-1 expression is controlled and driven by Nrf2 [49]. Antioxidant agents driving the upregulation of HO-1, such as dimethyl fumarate [50], gastrodin [51], and thioredoxin [52], have been reported beneficial in models of stroke and also reduce microglial activation and stroke-related neuroinflammation. Moreover, similar to our study, upregulation in HO-1 has been shown to be associated with reduced expression in COX-2 and NOS2 [53] and silencing HO-1 expression promotes LPS-induced pro-inflammatory response [54]. In the current study, HO-1 was expressed in both microglia/macrophages and neurons, and SSO was able to reverse LPS + IFNγ-induced downregulation of Nrf2 expression in microglia cultures. In fact, stimulation of the Nrf2 pathway has been shown to inhibit microglial activation [55, 56] supporting the anti-inflammatory capacity of Nrf2 pathway upregulation, our observations of the increased HO-1 expression in both microglia/macrophages and neurons, and reduced microgliosis and neuronal damage in SSO-treated ischemic mice. The fact that we did not observe an increase in HO-1 in SSO-treated BV2 cells may be related to the fact that in ischemic brains, crosstalk between microglia and neurons is likely to take place and both neurons and microglia contribute to the increased levels of HO-1. This indeed was evident by co-staining of HO-1 with NeuN and CD45 showing that both cell types express HO-1.

## Conclusions

Microglia play a prominent role in regulating stroke-induced inflammation. Microglia have been discussed to exacerbate brain damage following cerebral ischemia through the production of reactive nitrogen and oxygen species [4, 57, 58] but also secrete anti-inflammatory cytokines and aid in the process of inflammation resolution [27]. We have here shown for the first time that SSO is able to reduce the harmful microglial activation, promote the anti-oxidative response, and importantly reduce ischemic brain damage. Our in vitro data show that this is likely to be related to the inhibition of the p38 MAPK and NFκB pathways. Further studies are needed to determine the exact mechanism involved in SSO neuroprotection following ischemic stroke.

## Abbreviations

NFκB: Nuclear factor kappa-B
CBA: Cytokine bead array
COX-2: Cyclooxygenase-2
H3: Histone-3
HBSS: Hank’s Balanced Salt Solution
HO-1: Heme oxygenase-1
IFN-γ: Interferon-gamma
IL-1: Interleukin-1
IL-6: Interleukin-6
LPS: Lipopolysaccharide
MAPK: Mitogen-activated protein kinase
MCA: Middle cerebral artery
MCP-1: Monocyte chemoattractant protein-1
MRI: Magnetic resonance imaging
NO: Nitric oxide
Nrf2: Nuclear factor (erythroid-derived 2)-like 2
PBS: Phosphate buffer solution
PFA: Paraformaldehyde
pMCAo: Distal permanent occlusion of the MCA
SSO: Sulfosuccinimidyl oleate sodium
TLR: Toll-like receptor
TNF-α: Tumor necrosis factor-α
